# Study of the functionality of the *Helicobacter pylori trans*-translation components SmpB and SsrA in an heterologous system

**DOI:** 10.1186/1471-2180-10-91

**Published:** 2010-03-26

**Authors:** Marie Thibonnier, Sylvie Aubert, Chantal Ecobichon, Hilde De Reuse

**Affiliations:** 1 Institut Pasteur, Unité P. Pathogenèse de Helicobacter, 28 rue du Dr. Roux, 75724 Paris Cedex 15 France; 2 Current address: Institut Pasteur, Unité de Recherche et d'Expertise Bactéries anaérobies et Toxines, Paris, France; 3 Current address: Institut Pasteur, G5 Biologie et Génétique de la Paroi Bactérienne, Paris, France

## Abstract

**Background:**

*Trans*-translation is a ubiquitous bacterial quality control-mechanism for both transcription and translation. With its two major partners, SsrA a small stable RNA and the SmpB protein, it promotes the release of ribosomes stalled on defective mRNAs and directs the corresponding truncated proteins to degradation pathways. We have recently shown that *trans*-translation is an essential function in the gastric pathogen *Helicobacter pylori*. Our results suggested that some properties of the *H. pylori trans*-translation machinery distinguishes it from the well known system in *E. coli*. Therefore, we decided to test the functionality of the SmpB and SsrA molecules of *H. pylori *in the *E. coli *heterologous system using two established phenotypic tests.

**Results:**

*H. pylori *SmpB protein was found to successfully restore the *E. coli *Δ*smpB *mutant growth defect and its capacity to propagate λ*imm*^P22 ^phage. We showed that in *E. coli*, *H. pylori *SsrA (Hp-SsrA) was stably expressed and maturated and that this molecule could restore wild type growth to the *E. coli *Δ*ssrA *mutant. Hp-SsrA mutants affected in the ribosome rescue function were not able to restore normal growth to *E. coli *Δ*ssrA *supporting a major role of ribosome rescue in this phenotype. Surprisingly, Hp-SsrA did not restore the phage λ*imm*^P22 ^propagation capacity to the *E. coli *Δ*ssrA *mutant.

**Conclusions:**

These data suggest an additional role of the tag sequence that presents specific features in Hp-SsrA. Our interpretation is that a secondary role of protein tagging in phage propagation is revealed by heterologous complementation because ribosome rescue is less efficient. In conclusion, *tm*RNAs present in all eubacteria, have coevolved with the translational machinery of their host and possess specific determinants that can be revealed by heterologous complementation studies.

## Background

*Trans*-translation is a quality-control mechanism that is ubiquitous in bacteria and involves two activities [1-3]. First, *trans*-translation favors the rescue of ribosomes stalled on defective or damaged mRNAs (lacking a stop codon) through the restart of translation. Second, *trans*-translation functions to direct incomplete peptides to degradation by the addition of a specific tag [4]. *Trans*-translation is generally non-essential and requires two factors: SsrA, a small stable structured RNA (also called *tm*RNA) that acts both as a tRNA by its alanylated tRNA-like domain (TLD) and as a mRNA-like domain (MLD) [4] and its protein cofactor, SmpB.

The length and sequence of the *trans*-translation appended peptide tag varies with the bacterial species (between 8 and 35 amino acids) [5]. Mostly studied in *E. coli*, the tag encoded by SsrA is sufficiently informative to target any *trans*-translated proteins to degradation pathways [4]. The phenotypes of mutants deficient in this process depend on the species examined and are related to environmental adaptation, differentiation, stress response or virulence (for a review see [6]). Growing evidence indicates that *trans*-translation tagging targets specific substrates and therefore plays a regulatory role in organisms such as *Caulobacter crescentus *[7,8]*Yersinia pseudotuberculosis *[9], *Helicobacter pylori *[10] or *Streptomyces coelicolor *[11].

In *E. coli*, numerous phenotypes were associated with the deficiency of *trans*-translation, among which a slight enhancement of the doubling time that was observed even under normal growth conditions [12]. One of the tools used to characterize the SsrA determinants *in vivo *was the dependence on *trans*-translation of the growth of the hybrid bacteriophage λ*imm*^P22 ^in *E. coli *[13-15]. This phage is a hybrid between the *E. coli *lambda phage and the *Salmonella *P22 phage and is specific for *E. coli*. *E. coli *strains defective in *trans*-translation display a characteristic phenotype termed "Sip" (for selectively inhibits of λ*imm *^P22^) [13]. Indeed, the frequency of infection by λ*imm*^P22 ^is 10,000-fold lower in Δ*smpB *or Δ*ssrA E. coli *mutants as compared to that in the corresponding parental strain [13,16]. The precise molecular basis of the phage plating defect in *trans*-translation-deficient cells is not yet understood. The impact of SsrA point mutations on λ*imm*^P22 ^growth in *E. coli *was first analyzed by Withey and Friedman [14] who showed (i) that charging of *tm*RNA with Ala was essential and, (ii) that degradation of proteins tagged by *tm*RNA was only required to achieve optimal levels of phage growth. A more recent study challenged these conclusions and demonstrated that λ*imm*^P22 ^propagation in *E. coli *is exclusively dependent on ribosome recycling functions of *trans*-translation and not on its proteolysis targeting activity [15].

We have recently investigated the role of *trans*-translation in *Helicobacter pylori *[10]. *H. pylori *is a bacterial pathogen that colonizes the stomach of half of the human population and is strongly adapted to persist and multiply under stressful conditions such as low pH. Colonization of the stomach by *H. pylori *is associated with several gastric pathologies ranging from gastritis, peptic ulcer to adenocarcinoma [17]. We demonstrated that ribosome rescue by *trans*-translation is essential for *in vitro *growth of *H. pylori*. Interestingly, stress resistance and natural competence were strongly affected in *H. pylori *strains carrying a mutated *tm*RNA tag sequence [10]. While the overall structure of *H. pylori *SsrA is conserved, the tag sequence significantly differed from that of *E. coli *and our mutagenesis study revealed both identical and different properties as compared to its *E. coli *homolog [10]. To investigate further these differences using a model organism, we decided to study the *H. pylori *SmpB and SsrA expressed in the *E. coli *heterologous system.

## Results

### Functional complementation of an *E. coli smpB *deletion mutant by Hp-SmpB

**Figure 1:**
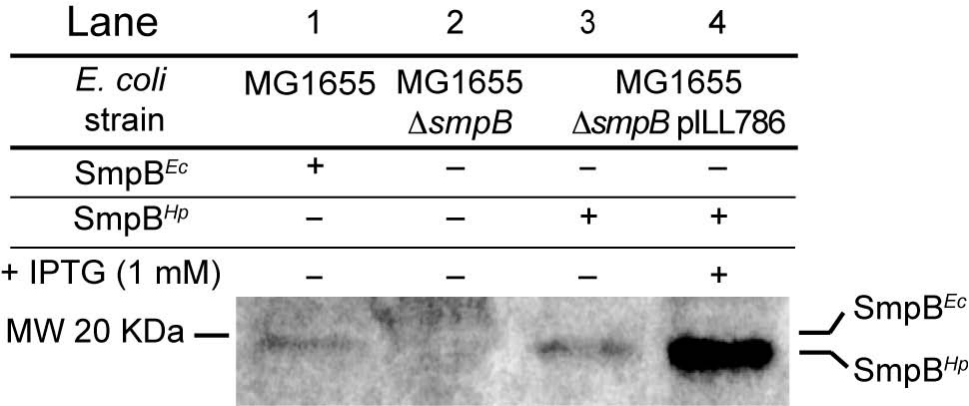
**Detection of SmpB in *E. coli***. Detection of SmpB protein in *E. coli *was performed by western blot with an *E. coli *SmpB polyclonal antibody. Lane 1: wild type *E. coli *strain (predicted MW SmpB^*Ec *^= 18,125 Da), lane 2: Δ*smpB E. coli *mutant. Lanes 3-4: SmpB^*Hp *^detection in a Δ*smpB E. coli *mutant carrying the inducible vector pILL786 expressing the *smpB*^*Hp *^gene (predicted MW SmpB^*Hp *^= 17,682 Da), with or without induction with 1 mM IPTG, respectively. Calibrated amounts of crude bacterial extracts were separated by SDS-15% PAGE. MW: molecular weight.

To examine the functionality of the SmpB protein of *H. pylori *(Hp-SmpB) in *E. coli*, the corresponding gene *hp1444 *was amplified from *H. pylori *strain 26695 and cloned into pILL2150 under control of an inducible promoter, to generate pILL786 (Table 1). This plasmid was transformed into *E. coli *wild type strain MG1655 and its isogenic *ΔsmpB *mutant [18] (Table 1 and 2). Expression of Hp-SmpB in *E. coli *was verified by western blot in the *ΔsmpB *mutant using antibodies raised against purified *E.coli *SmpB. Hp-SmpB was detected, its synthesis was strongly enhanced upon addition of IPTG and was over-expressed in comparison with the *E. coli *endogenous SmpB protein, Ec-SmpB (Figure 1).

**Table 1:** Plasmids used in this study

Plasmids	Relevant features	Reference
pEXT21	low copy number *E. coli *vector	[25]

pILL2318	*H. pylori ssrA*^*WT *^cloned into pEXT21	This study

pILL2150	high copy number *H. pylori*/*E. coli *shuttle vector	[24]

pILL2334	*E. coli ssrA*^*WT *^cloned into pILL2150	This study

pILL786	*hp1444 *encoding Hp-SmpB cloned into pILL2150	This study

pILL788	*H. pylori ssrA*^*WT *^cloned into pILL2150	[10]

pILL791	*H. pylori ssrA*^*DD *^cloned into pILL2150	[10]

pILL792	*H. pylori ssrA*^*resume *^cloned into pILL2150	[10]

pILL793	*H. pylori ssrA*^*wobble *^cloned into pILL2150	[10]

pILL794	*H. pylori ssrA*^*SmpB *^cloned into pILL2150	[10]

pILL2328	*H. pylori ssrA*^*STOP *^cloned into pILL2150	[10]

**Table 2:** *E. coli *strain used in this study.

Strains	*ssrA *and *smpB *alleles	Plasmids [antibiotic resistance]
MG1655 pILL2150	*smpB*^*Ec *^*ssrA*^*Ec*^/pILL2150	multicopy [Cm]

MG1655 pEXT21	*smpB^Ec ^ssrA^Ec^*/pEXT21>	low copy [Sp]

MG1655 Δ*smpB *pILL2150	Δ*smpB^Ec ^ssrA^Ec^*/pILL2150	multicopy [Cm]

MG1655 Δ*smpB *pILL786	Δ*smpB^Ec ^ssrA^Ec^*/pILL2150 with *smpB^Hp^*	multicopy [Cm]

MG1655 Δ*ssrA *pILL2150	*smpB*^*Ec*^Δ*ssrA*^*Ec*^/pILL2150	multicopy [Cm]

MG1655 Δ*ssrA *pILL2334	*smpB^Ec^*Δ*ssrA^Ec^*/pILL2334 with *ssrA^Ec-WT^*	multicopy [Cm]

MG1655 Δ*ssrA *pILL788	*smpB^Ec^*Δ*ssrA^Ec^*/pILL2150 with *ssrA^Hp-WT^*	multicopy [Cm]

MG1655 Δ*ssrA *pILL2318	*smpB^Ec^*Δ*ssrA^Ec^*/pEXT21 with *ssrA^Hp-WT^*	low copy [Sp]

MG1655 Δ*ssrA *pILL791	*smpB^Ec^*Δ*ssrA^Ec^*/pILL2150 with *ssrA^Hp-DD^*	multicopy [Cm]

MG1655 Δ*ssrA *pILL792	*smpB^Ec^*Δ*ssrA^Ec^*/pILL2150 with *ssrA^Hp-resume^*	multicopy [Cm]

MG1655 Δ*ssrA *pILL793	smpB^Ec^ΔssrA^Ec^/pILL2150 with ssrA^Hp-wobble^	multicopy [Cm]

MG1655 Δ*ssrA *pILL794	*smpB^Ec^*Δ*ssrA^Ec^*/pILL2150 with *ssrA^Hp-smpB^*	multicopy [Cm]

MG1655 Δ*ssrA *pILL2328	*smpB^Ec^*Δ*ssrA^Ec^*/pILL2150 with *ssrA^Hp-STOP^*	multicopy [Cm]

The doubling time of each *E. coli *strain was calculated from growth curves performed in LB medium at 37°C with chloramphenicol [Cm] 100 μg/ml or with spectinomycin [Sp] 100 μg/ml.

**Figure 2:**
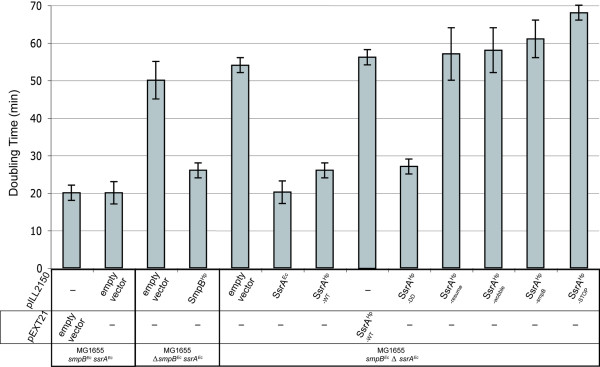
**Doubling time of *E. coli ΔssrA *or *ΔsmpB *mutants expressing SmpB^*Hp *^WT, SsrA^*Hp *^WT or mutants**. Doubling times were calculated for *E. coli *strains expressing SmpB^*Hp*^, SsrA^*Hp *^and different mutant versions of SsrA^*Hp *^from plasmids. Doubling times (g values) correspond to the mean generation time. As a control, growth complementation of the *E. coli ΔssrA *with *Ec-ssrA *is presented. Empty vector corresponds to a vector without insert.

The efficacy of propagation of the hybrid phage λ*imm*^P22 ^[13] was measured on different strains. Table 3 presents the relative efficiency of plating (EOP) of each strain in comparison with that of the wild type parental strain. Phage propagation on strain MG1655 *ΔsmpB *containing the empty vector pILL2150 was, as expected, strongly affected with an EOP of 1.3 × 10^-5 ^(Table 3). Relative EOP of strain MG1655 *ΔsmpB *pILL786 in the presence of IPTG, expressing Hp-SmpB is close to 1 (Table 3). This result demonstrated that Hp-SmpB is active in *E. coli *and efficiently complemented the phage propagation defect phenotype. In addition, the growth defect of MG1655 *ΔsmpB *mutant was analyzed with or without Hp-SmpB. Under our test conditions, MG1655 Δ*smpB *mutant presented a doubling time that was about twice that of the wild type strain and was restored to wild type growth in the presence of Hp-SmpB expressed by pILL786 (Figure 2 and Table 3). This indicated that Hp-SmpB is able to replace Ec-SmpB functions during *trans*-translation in *E. coli*.

**Table 3:** Ability of *H. pylori *SmpB and of wild type or mutant alleles of *ssrA*^*Hp *^to support growth of λ*imm*^P22 ^in *E. coli *Δ*ssrA *or Δ*smpB *deletion mutants and to restore the growth defect in *E. coli *Δ*ssrA or *Δ*smpB *mutants

Strains	*ssrA *or *smpB *alleles	EOP^§^	Growth defect restoration in *E. coli *Δ*smpB *or in *E. coli *Δ*ssrA*	
MG1655	*smpB^Ec ^ssrA^Ec^*	1	-	

MG1655 Δ*smpB *pILL2150	Δ*smpB^Ec ^ssrA^Ec^*	1.3 × 10^-5^	no	

MG1655 Δ*smpB *pILL786	Δ*smpB^Ec ^ssrA^Ec^/smpB^Hp^*	0.6	yes	

MG1655 Δ*ssrA *pILL2150	*smpB^Ec^*Δ*ssrA^Ec^*	2.6 × 10^-5^	no	

MG1655 Δ*ssrA *pILL2334	*smpB^Ec ^*Δ*ssrA^Ec^/ssrA^Ec-WT^*	1	yes	

MG1655 Δ*ssrA *pILL788	*smpB^Ec ^*Δ*ssrA^Ec^/ssrA^Hp-WT^*	5.0 × 10^-5^	yes	

MG1655 Δ*ssrA *pILL791	*smpB^Ec ^*Δ*ssrA^Ec^/ssrA^Hp-DD^*	1.6 × 10^-5^	yes	

MG1655 Δ*ssrA *pILL2328	*smpB^Ec ^*Δ*ssrA^Ec^/ssrA^Hp-STOP^*	6.1 × 10^-5^	no	

MG1655 Δ*ssrA *pILL792	*smpB^Ec ^*Δ*ssrA^Ec^/ssrA^Hp-resume^*	3.9 × 10^-5^	no	

MG1655 Δ*ssrA *pILL793	*smpB^Ec ^*Δ*ssrA^Ec^/ssrA^Hp-wobble^*	2.3 × 10^-5^	no	

MG1655 Δ*ssrA *pILL794	*smpB^Ec ^*Δ*ssrA^Ec^/ssrA^Hp-smpB^*	3.6 × 10^-5^	No	

^§ ^EOP is the ratio of the titer of phage on a lawn of bacteria mentioned in the table divided by the titer of phage on a wild type bacterial lawn.

### Expression and maturation of Hp-SsrA in *E. coli*

**Figure 3:**
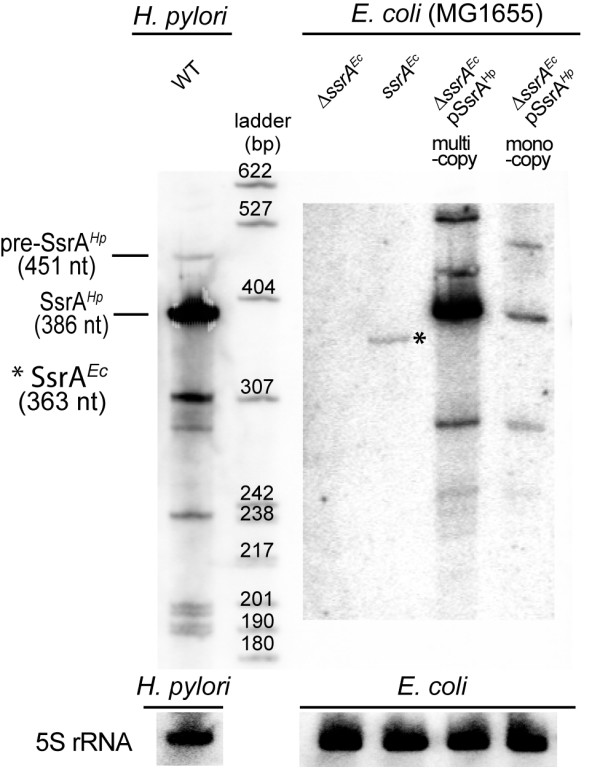
**Detection of SsrA^*Hp *^expressed in *H. pylori *and from plasmids in *E. coli***. A SsrA^*Hp *^riboprobe was used to perform northern blots and detect the SsrA^*Hp *^molecule in *H. pylori *and in *E. coli *wild type or *ΔssrA *mutant strains. Pre-SsrA^*Hp *^indicates a band with the size of non-maturated precursor of SsrA^*Hp*^. A faint band marked by a star corresponds to cross-hybridization with the SsrA^*Ec *^that is, as expected, absent in the *E. coli ΔssrA *mutant.

To evaluate the heterologous complementation capacity of Hp-SsrA in *E. coli*, we constructed pILL788 and pILL2318 carrying the *ssrA *gene of *H. pylori *under control of a promoter on high copy and low copy number plasmids, respectively (Table 1). Plasmids pILL788 and pILL2318 expressing wild type Hp-SsrA were transformed into both MG1655 wild type and Δ*ssrA *strains (Table 2). The expression of Hp-SsrA was examined by northern blot with total RNA extracted from different *E. coli *strains and from the *H. pylori *26695 strain (Figure 3). A 300 nt long riboprobe was chosen in the region of Hp-SsrA displaying homology with Ec-SsrA. A band of 386 nt that matches the size of the mature Hp-SsrA was detected in the RNA samples extracted from *E. coli *MG1655 Δ*ssrA *pILL788 and MG1655 Δ*ssrA *pILL2318 strains (Figure 3). As expected, the amount of Hp-SsrA is weaker when expressed from the low copy plasmid pILL2318 than from pILL788. With RNA extracted from *H. pylori *strain 26695, we observed an intense band of the same size that was absent in samples extracted from MG1655 Δ*ssrA *containing pILL2150, the empty vector (Figure 3). A faint band corresponding to mature Ec-SsrA (363 nt) was detected in *E. coli *MG1655 wild type strain. This indicates that in *E. coli*, Hp-SsrA is expressed and correctly maturated.

### Analysis of the functionality of Hp-SsrA in *E. coli*

The capacity of Hp-SsrA to complement the phage propagation defect of an *E. coli *strain deficient in SsrA was examined. The EOP of strain MG1655 Δ*ssrA *pILL2150 (empty vector) was 2.6 × 10^-5 ^as expected (Table 3). Surprisingly, the presence of pILL788 expressing processed Hp-SsrA in strain MG1655 Δ*ssrA *did not restore the capacity to propagate phage λ*imm*^P22 ^(Table 3). This showed that Hp-SsrA is not able to replace Ec-SsrA in this phenotypic test. It was controlled that phage λ*imm*^P22 ^propagation was restored in strain MG1655 Δ*ssrA *pILL2334 expressing wild type Ec-SsrA on a plasmid.

Under our test conditions, the doubling time of *E. coli ΔssrA *mutant was twice that of the wild type strain (Figure 2). Interestingly, wild type growth was restored in the *E. coli *Δ*ssrA *mutant complemented with plasmid pILL788 that expresses high levels of Hp-SsrA (Figure 2) but not with plasmid pILL2318 that expresses low levels of Hp-SsrA. As a control, wild type growth was also observed with strain MG1655 Δ*ssrA *pILL2334 expressing wild type Ec-SsrA. This indicated that Hp-SsrA is functional to rescue the growth defect of *E coli *Δ*ssrA *but is not able to restore the phage propagation deficiency. We then wanted to understand further the functional basis of the partial functionality of Hp-SsrA in *E. coli*.

### Analysis of the functionality of mutated Hp-SsrA versions in *E. coli*

**Figure 4:**
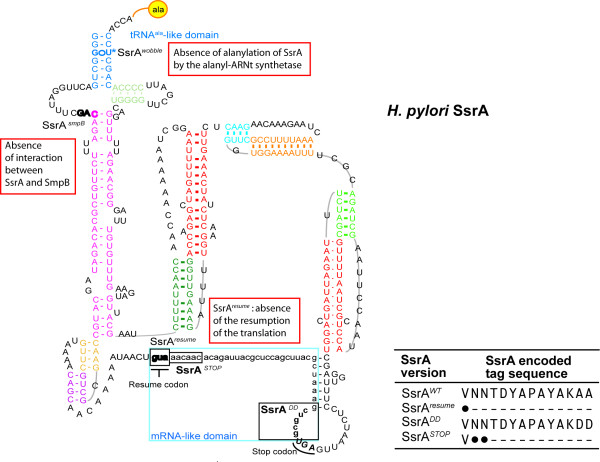
**Mutations introduced into the *H. pylori* SsrA molecule**. The model of the *H. pylori *mature SsrA molecule is after the *tm*RNA website http://www.indiana.edu/~tmrna/. As described in [10], the SsrA^*wobble*^, SsrA^*SmpB*^, SsrA^*resume *^mutations that abolish the *trans-*translation process are boxed in red. Mutations of the mRNA-like domain that affect the tag are also indicated. The amino acid sequence of the tag (wild type or mutant) appended to *trans*-translated proteins are listed in the table.

In a previous study, we constructed a series of five *H. pylori *SsrA mutants and evaluated in *H. pylori *their impact on *trans*-translation, survival and stress-response [10]. Characteristics of these mutations are summarized in Figure 4. Plasmids pILL793, pILL794 and pILL792 express mutant Hp-SsrA that are unable to be alanylated on the TLD (SsrA^wobble^), to interact with SmpB (SsrA^SmpB^) and to restart the translation on the MLD (SsrA^resume^), respectively. Each of this mutation was found to be essential for growth of *H. pylori *[10]. When these plasmids were tested for complementation of the *E. coli *Δ*ssrA *mutant, neither phage propagation nor growth defective phenotypes was rescued (Figure 2 and Table 3).

In *H. pylori*, two mutations in the MLD of Hp-SsrA were found to be viable but affected the capacity of the corresponding mutant strains to resist to various stresses [10]. One mutation targets the terminal part of the tag sequence, the corresponding mutant gene Hp-SsrA^DD ^is carried by plasmid pILL791. This mutation was chosen because it was described to stabilize the *trans*-translated proteins in species like *E. coli*. In another mutant, Hp-SsrA^STOP ^(carried by pILL2328) two stop codons were introduced immediately downstream from the resume codon. As a consequence, Hp-SsrA^STOP ^adds a minimal tag (Ala-Val) to *trans*-translated proteins (Figure 4).

These two mutated Hp-SsrA versions did not restore the phage propagation capacity to the *E. coli ΔssrA *mutant (Table 3). Interestingly, growth defect of the *E. coli ΔssrA *mutant was restored to the wild type level by complementation with pILL791 expressing Hp-SsrA^DD^, and not with pILL2328 expressing Hp-SsrA^STOP^.

## Discussion

*Trans*-translation is a bacterial ubiquitous mechanism of quality-control for protein and mRNA synthesis. We have recently shown that *trans*-translation is essential for *in vitro *growth of the gastric pathogen *H. pylori *[10] like in a few other human pathogens, *Mycoplasma genitalium *[19], *Neisseria gonorrhoeae *[20] or *Haemophilus influenzae *[21]. We also demonstrated that in *H. pylori*, the essential *trans*-translation function is ribosome rescue and that a single ribosomal translocation step is sufficient to promote release of stalled ribosomes [10]. Using different mutants of *H. pylori ssrA*, we found that under conditions of functional ribosome rescue, the tagging of *trans*-translated proteins was required for tolerance to both oxidative and antibiotic stresses and for effective natural competence. These data revealed for the first time that control of protein degradation through *trans*-translation is by itself central in the management of stress conditions and of competence and supports a regulatory role of *trans*-translation dependent protein tagging. Since we anticipate that this regulatory role of protein tagging is underestimated in *E. coli *and because we possessed a collection of well-defined Hp-SsrA mutant, we decided to explore the functionality of the *H. pylori trans*-translational components in *E. coli*.

Measurement of the λ*imm*^P22 ^phage propagation is a classical test to evaluate the functionality of *trans*-translation in *E. coli*. As previously reported, both Δ*ssrA *and Δ*smpB E. coli *mutants exhibit a 10,000-fold defect of phage propagation [14]. *E. coli *SsrA mutants present a slight growth defect, enhanced sensitivity to stress and to sub-inhibitory antibiotic concentrations. These phenotypes are complemented by *E. coli *SsrA variants that add a tag lacking some proteolytic determinants (f.i SsrA^DD^). Therefore, these phenotypes are likely not to depend on proteolysis.

In a first test, *H. pylori *SmpB protein was found to successfully complement the *E. coli *Δ*smpB *mutant for both phage propagation and growth despite only 34.6% identity between Ec-SmpB and Hp-SmpB. This showed that Hp-SmpB is able to interact with both the *E. coli *SsrA RNA and ribosomes to perform efficient *trans*-translation in *E. coli*.

Results with Hp-ssrA in *E. coli *revealed a more complex picture. First, we showed that upon expression in *E. coli*, Hp-SsrA is highly expressed and exhibits a size compatible with correct maturation. Indeed, Hp-SsrA and Hp-SsrA^DD ^restored a wild-type growth phenotype to an *E. coli *Δ*ssrA *mutant indicating its functionality in *E. coli*. This result is in agreement with a minor role of the protein tagging step in the growth defect of *Ecoli *Δ*ssrA*. Accordingly, we observed that the mutant versions of Hp-SsrA that were affected in ribosome rescue (SsrA^Resume^, SsrA^wobble ^and SsrA^SmpB^) failed to complement the slow growth phenotype of *E. coli *Δ*ssrA*. Unexpectedly, the Hp-SsrA^STOP ^mutant that contains an intact resume codon followed by two stop codons is not able to complement the *E. coli *Δ*ssrA *growth defect. This is surprising since in *H. pylori*, the SsrA^STOP ^mutation is not essential for *in vitro *growth strongly suggesting that it is still effective in release of stalled ribosomes [10]. In a previous study [15], an equivalent mutation was introduced into *E. coli *SsrA, however only phage propagation phenotype is reported and no mention was made of the growth rate of this mutant. The most straightforward interpretation of our data is that *trans*-translation by Hp-SsrA^STOP ^in *E. coli *is not efficiently using the resume codon. Indeed, there are striking differences between Hp-SsrA and Ec-SsrA. In particular, the resume codon of Hp-SsrA is GUA encoding Valine and in *E. coli*, the resume codon GCA encodes Alanine (Figure 4) [5]. Replacement of the Ec-SsrA resume codon by GUA or GUC encoding Valine is functional in *E. coli *[22]. However, mass spectrometry analysis revealed that breakage of the peptide tag occurred frequently after certain residues like a Valine encoded by GUA and that these SsrA-tag added to proteins are ineffective in growth competition with *ΔssrA *mutants [22]. Therefore, we hypothesize that the GUA resume codon of Hp-SsrA is a poor resume codon for *trans-*translation in *E. coli *and that additional downstream sequence compensate for this deficiency. As a consequence, the introduction of two stops immediately after the resume codon as in the Hp-SsrA^STOP ^mutant might render this compensation impossible and translation restart ineffective. These data emphasize the strict constraints on SsrA sequence to achieve ribosome rescue in a given organism.

The functionality of Hp-SsrA in *E. coli *was also examined using the phage λ*imm*^P22 ^propagation test. Several studies illustrated in Table 4 conclude that λ*imm*^P22 ^propagation in *E. coli *is mainly dependent on efficient ribosome rescue and that the inactivation of the tagging activity did not affect phage growth. It was also reported that the threshold SsrA function required for plaque formation in *E. coli *is fairly low [23]. Thus, the absence of phage λ*imm*^P22 ^propagation in the *E. coli *Δ*ssrA *expressing wild type Hp-SsrA (that complements growth defect) was unexpected (Table 3). In contrast to Hp-SsrA, wild-type SsrA from *Neisseria gonorrhoeae *(NG-SsrA) restores phage propagation in *E. coli *Δ*ssrA *[20]. Interestingly, NG-SsrA mutant versions carrying mutations affecting either the ribosome rescue function (NG-SsrA^UG^) or the functionality of the tag sequence (SsrA^DD ^and SsrA^Ochre^) were defective in complementing the phage propagation in *E. coli *Δ*ssrA*. This suggests that under conditions of heterologous complementation of *E. coli *Δ*ssrA *either with Hp-SsrA (this work) or with NG-SsrA [20], λ*imm*^P22 ^phage propagation requires *trans*-translation-dependent protein tagging in addition to ribosome rescue. The proposition of a secondary role of protein tagging in λ*imm*^P22 ^propagation in *E. coli *is compatible with the observation by Withey and Friedman [14] that smaller plaques were generated in an *E. coli *strain expressing a SsrA^0 ^mutant that encodes a truncated tag. They postulate that the tag is not necessary for phage propagation but is required to allow an optimal growth of phages.

**Table 4:** Phenotypes of the different mutants of *E. coli ssrA*

E. coli SsrA version	Effects on SsrA	SsrA tag appended to truncated proteins		EOP^§^	Reference
SsrA^WT^	Wild type	ANDENYALAA		1	[14,15]

SsrA^resume^	Substitution of the resume codon by a stop codon	None		1.3 × 10^-5^	[14]

SsrA^wobble^	Absence of alanylation of the tRNA-like domain of SsrA	None		5 × 10^-5^	[28]

SsrA^SmpB^	Absence of interaction between SsrA and SmpB	None		N.D.	

SsrA^DD^	Substitution of the last two alanine residues of the tag by two aspartate residues	ANDENYALDD		0.5 -- 0.1	[28]

SsrA^STOP^	Two stop codons added after the resume codon	Minimal tag added		0.9	[14]

^§ ^EOP is the ratio between the titer of phage on a lawn of bacteria expressing one of the indicated SsrA versions and the titer of phage on a wild type bacterial lawn; N.D.: Not determined.

## Conclusions

To conclude, heterologous complementation showed that the wild type Hp-SsrA is able to restore normal growth to an *E. coli ΔssrA *mutant suggesting that despite the sequence differences between these molecules, Hp-SsrA acts as a partially functional but not optimal *tm*RNA in *E. coli*. The tag sequence of Hp-SsrA presents several differences with that of the other studied bacteria, in particular a different resume codon, a charged residue at the end of the tag (Lysine instead of Leucine or Valine) (Figure 4) and the absence of a SspB protein recognition motif. We propose that these differences might account for the inability of the Hp-SsrA to support phage propagation in an *E. coli ΔssrA *mutant. This attributes an additional role of *trans*-translational dependent tagging for efficient λ imm^P22 ^phage propagation in *E. coli*. Our interpretation is that this secondary role of protein tagging is revealed by heterologous complementation because ribosome rescue is less efficient. This emphasizes once again the regulatory role of *trans*-translation in addition to its quality control function.

In conclusion, *tm*RNAs found in all eubacteria, have coevolved with the translational machinery of their host and possess specific determinants that were revealed by this heterologous complementation study.

## Methods

### Bacterial strains and growth conditions

*Escherichia coli *strain MG1655, MG1655 Δ*ssrA *[18] and MG1655 Δ*smpB *[18] were grown at 37°C on solid or liquid LB medium. These strains were used as recipients for plasmids carrying different *H. pylori *genes:*smpB, ssrA *and mutant versions of *ssrA *as well as the *E. coli ssrA *gene (Table 2). Both antibiotics chloramphenicol (Cm) and spectinomycin (Sp) were used at 100 μg ml^-1 ^and isopropyl-β-D-thiogalactoside (IPTG) at 1 mM. *H. pylori *strain 26695 was grown under standard conditions, and harvested in mid-log phase as described in [10]. Doubling times (g values) correspond to the mean generation time.

### Molecular techniques and sequencing

Plasmids pILL788, pILL791, pILL792, pILL793, pILL794, pILL795, pILL2328 correspond to *H. pylori ssrA*^*WT*^, *ssrA*^*DD*^, *ssrA*^*resume*^, *ssrA*^*wobble*^, *ssrA*^*smpB*^, *ssrA*^*STOP *^genes cloned into the *E. coli*/*H. pylori *shuttle vector pILL2150 [24], respectively. SsrA mutagenesis has been described in [10]. The *H. pylori ssrA *gene amplified by PCR with primers H367 (5'-CG*GGATCC*CTCACCTGTTCTTTCTGA-3') and H368 (5'-GG*GGTACC*CGGATCCTT AATCGAATAAAAATCAGG-3') was cloned into the pEXT21 low copy number vector (1-3 copies per cell) [25] using *Bam*HI/*Kpn*I restriction sites (Table 1). The resulting plasmid was designated pILL2318.

The *E. coli ssrA *gene amplified by PCR with primers H365 5'-CTATCCCGGCGC TGGGTAACATCGGG-3, and H366 5'-GCTTTTCGTTGGGCCTATCAATGGGCC-3' was cloned into pILL2150, to generate pILL2334. The *H. pylori smpB *gene amplified by PCR with primers H225 (5'-GGACTAGTAGGAAGAGAATAATGAAACTCATTGCCAG CAAC-3') and H236 (5'-CGGGGTACCTTATCCTTTAAAGTGGTGTTTTAAATCAGC-3'), was cloned into pILL2150 [24] using *Spe*I/*Kpn*I restriction sites to generate pILL786.

### Test of λimm^P22 ^propagation in *E. coli*

The efficiency of plating (EOP) strains was determined by plating tenfold serial dilution of phage λ*imm*^P22 ^on top agar mixed with 100 μl *E. coli *overnight liquid culture in LB with 0.4% maltose and 10 mM MgSO_4_. The number of CFU·ml^-1 ^was calculated for each *E. coli *strain. The EOP is the ratio between the titer of phage on a bacterial lawn of the indicated strain (Table 3) and that of the wild type strain.

### Western blot

Western blot to detect SmpB proteins was performed with *E. coli *whole cell sonicates prepared as in [26]. Protein concentrations were measured with Bradford assay (Bio-Rad). Twenty μg of crude extracts were separated by 15% SDS-PAGE and blotted on a polyvinylidene difluororide membrane (PVDF, Millipore). Hp-SmpB and Ec-SpmB were detected with rabbit polyclonal antibody raised against Ec-SmpB (a generous gift of B. Felden). Binding of the IgG anti-rabbit coupled peroxydase antibody (Amersham) was revealed with the ECL Plus reagent (Pierce).

### RNA extraction, riboprobe synthesis and northern blot

RNAs were extracted using the phenol-chloroform method as described in [27]. An *E. coli *5S rRNA riboprobe was synthesized using both primers H357 (5-GCCTGGCGGCAGTAGCG CG GTGG-3') and H358 (5'-CTAATACGACTCACTATAGGGAGAGCCTGGCAGTTCCC TACTCTCGC-3'). Riboprobes synthesis for *H. pylori *SsrA was as in [10]. The ladder used corresponds to pBR322 vector digested by *Msp*I and labeled at the 5'end with γ ^32^P ATP. Intensities of the bands were determined with Quantity One Software (Bio-Rad). The northern blot procedure was as described in [10].

## Authors' contributions

Conceived and designed the experiments: MT, HDR. Performed the experiments: MT, SA, CE. Analyzed the data: MT, HDR. Wrote the paper: MT, HDR. All authors read and approved the final manuscript.
